# An Acute, Placebo-Controlled, Single-Blind, Crossover, Dose-Response, Exploratory Study to Assess the Effects of New Zealand Pine Bark Extract (Enzogenol^®^) on Glycaemic Responses in Healthy Participants

**DOI:** 10.3390/nu12020497

**Published:** 2020-02-15

**Authors:** Wen Xin Janice Lim, Lynne Chepulis, Pamela von Hurst, Cheryl S. Gammon, Rachel A. Page

**Affiliations:** 1 School of Health Sciences, Massey University, Auckland 0632, New Zealand; w.x.j.lim@massey.ac.nz (W.X.J.L.); C.Gammon@massey.ac.nz (C.S.G.); 2 Waikato Medical Research Centre, University of Waikato, Hamilton 3216, New Zealand; lynnec@waikato.ac.nz; 3 School of Sport, Exercise and Nutrition, Massey University, Auckland 0632, New Zealand; P.R.vonHurst@massey.ac.nz; 4 School of Health Sciences, Massey University, Wellington 6021, New Zealand; 5 Centre for Metabolic Health Research, Massey University, Auckland 0632, New Zealand

**Keywords:** New Zealand pine bark extract, Enzogenol^®^, proanthocyanidins, impaired glycaemic control, hypoglycaemic effects

## Abstract

An acute, placebo-controlled, single-blind, crossover, dose-response, exploratory study was designed to investigate the hypoglycaemic effects of New Zealand pine bark extract (Enzogenol^®^). Twenty-five healthy participants categorised into having a monophasic or complex (biphasic or triphasic) glucose curve shape at the control visit consumed a placebo and Enzogenol^®^ (50 and 400 mg) on three separate occasions before an oral glucose tolerance test (OGTT). In the monophasic group, 50 and 400 mg of Enzogenol^®^ significantly reduced the mean glucose incremental area under the curve (iAUC) compared to control 241.3 ± 20.2 vs. 335.4 ± 34.0 mmol/L·min, *p* = 0.034 and 249.3 ± 25.4 vs. 353.6 ± 31.5 mmol/L·min, *p* = 0.012, respectively. The 400 mg dose further reduced the percentage increment of postprandial glucose (%PG) 31.4% ± 7.9% vs. 47.5% ± 8.6%, *p* = 0.010, glucose peak 7.9 ± 0.3 vs. 8.9 ± 0.3 mmol/L, *p* = 0.025 and 2h-OGTT postprandial glucose (2hPG) 6.1 ± 0.3 vs. 6.7 ± 0.3 mmol/L, *p* = 0.027. Glucose iAUC was not significantly different in the complex group, except for reductions in %PG 28.7% ± 8.2% vs. 43.4% ± 5.9%, *p* = 0.012 after 50 mg dose and 27.7% ± 5.4% vs. 47.3% ± 7.2%, *p* = 0.025 after 400 mg dose. The results suggest that Enzogenol^®^ may have hypoglycaemic effects in healthy participants, especially those exhibiting monophasic shapes.

## 1. Introduction

Type 2 diabetes mellitus (T2DM) is characterised by abnormally high blood glucose levels otherwise termed as hyperglycaemia [1]. It accounts for over 90% of diabetes diagnoses [2], and can result in significant morbidity and mortality from macrovascular and microvascular complications, such as retinopathy, nephropathy, and neuropathy [3,4,5].

Natural plant extracts have been increasingly explored as options to treat and manage impaired glycaemic control [6,7,8,9]. They are seen as a potential alternative to anti-diabetic medications that are associated with a range of adverse effects, including weight gain, risk of hypoglycaemia, and gastrointestinal discomfort [10]. One such natural extract is food-grade pine bark, obtained from the bark of pine trees in timber industries that otherwise would be discarded [11,12]. Recent studies examining various sources of pine bark extracts have shown that pine bark possessed antioxidant properties, anti-inflammatory, neuro-protective, and anti-diabetic effects [11,13,14].

Naturally occurring polyphenols have been suggested to play a major role in the anti-diabetic properties exhibited by pine bark extracts, and proanthocyanidins are the main and most abundant bioactive components [11,12,15,16]. The New Zealand pine bark extract, trade name Enzogenol^®^, is produced from *Pinus radiata* trees grown in New Zealand by a water-based extraction [11,12]. The dry powder contains greater than 80% proanthocyanidins, 1–2% taxifolin, other flavonoids and phenolic acids, and some carbohydrates [12]. The proanthocyanidin content in Enzogenol^®^ was also shown to be even higher than Pcynogenol^®^, a French Maritime pine bark produced from the outer bark of *Pinus pinaster* Ait. Subsp. *Atlantica* growing in the Southwest coastal region in France [16,17].

There is increasing evidence showing that healthy individuals with normal glucose tolerance (NGT) may still eventually develop T2DM [18,19,20,21,22] depending on their patterns of postprandial glucose [23,24,25,26,27,28]. It is suggested that 20% of NGT individuals already have a certain degree of insulin resistance [25,29,30,31], although their postprandial blood glucose may fall within the normal range.

Patterns of postprandial glucose obtained from the 2h oral glucose tolerance test (OGTT) have been extensively used in research to predict future T2DM in NGT individuals before its onset [32,33]. Studies have shown that individuals with monophasic glucose curve shapes during a 2h OGTT, defined by having only one peak in the glucose curve, tended to have a heightened risk of T2DM [23,24,25,26,27,28]. On the other hand, those exhibiting biphasic or triphasic (complex) glucose curve shapes, defined by having more than one peak in the glucose curve, are more likely to have a lower risk of T2DM [23,24,25,26,27,28]. Cross-sectional and longitudinal studies have observed that NGT individuals possessing monophasic glucose curve shapes, in contrast to those having complex shapes, tended to have a significantly higher glucose, insulin, C-peptide, free fatty acid, and visceral fat [24,25,26], with significantly reduced insulin sensitivity, a lack of compensatory first and second phase insulin secretion, and higher levels of insulin resistance [23,24,25,27,33].

Clinical studies have also revealed that depending on the degree of glucose tolerance in individuals defined by various glucose response indices including glucose curve shapes, they may either be responders or non-responders to a given intervention such as with natural plant extracts [34,35,36,37]. Krishnan and colleagues have also emphasised the importance of examining dietary interventions using an integrated physiological approach that stratified participants with varying glycaemic responses into subgroups in contrast to overall group responses [37].

To date, no human study has been conducted on the hypoglycaemic effects of Enzogenol^®^ that was based on subgroup glycaemic responses to the intervention given. Hence, this present study was an exploratory study to first investigate the acute, dose-dependent, hypoglycaemic potential of Enzogenol^®^ on healthy, NGT participants stratified into subgroups of glycaemic responses. The subgroups were based on their postprandial glucose curve shapes (monophasic vs. complex) after an OGTT at the control visit. Further to this, by stratifying participants according to their postprandial glucose curve shapes, we aimed to investigate if there was a difference in treatment outcome and dose response to Enzogenol^®^ between the two groups. The study examined both the primary outcome measurement of glucose incremental area under the curve (iAUC) and secondary changes in glycaemic response indices, such as the percentage increment of postprandial glucose (%PG), the time to glucose peak, the glucose peak value, and 2h postprandial glucose at 120 min of OGTT (2hPG), to enhance the understanding of the potential hypoglycaemic effects of Enzogenol^®^. Any observed acute effects from this preliminary intervention with Enzogenol^®^ in healthy population would help inform future studies that include investigating the impact of Enzogenol^®^ over at least 8–12 weeks in both normo- and hyperglycaemic participants with worsening glucose intolerance, such as those with prediabetes or T2DM. Additionally, future studies can determine the effects of Enzogenol^®^ on insulin secretion and sensitivity, as well as beta-cell function (e.g., HOMA-IR, Matsuda index, and other measures), and explore the mechanistic action of Enzogenol^®^ in improving blood glucose responses.

## 2. Methods

### 2.1. Study Population

The study was approved in March 2018 by the Massey University Human Ethics Committee (MUHEC) (ref: SOA 17/73). The clinical trial was registered retrospectively at anzctr.org.au (Australia New Zealand Clinical Trials Registry Number: ACTRN12619001571167). The study was conducted in accordance with the Declaration of Helsinki and all participants gave their informed consent prior to participating in the study.

Participants were recruited from Auckland, New Zealand using poster advertisements within the local university and community (March–December 2018). They were selected according to the following inclusion criteria: (i) healthy Body Mass Index (BMI) of 18.5–25.0 kg/m^2^, (ii) aged 18–40 years, (iii) not suffering from any impaired glycaemic control (fasting blood glucose (FPG) < 5.5 mmol/L) and glycated haemoglobin A1c (HbA1c) < 40 mmol/mol (Cobas b 101 HbA1c test, CV 0.8–1.7%, Roche Diagnostics), (iv) not taking any forms of glucose-lowering medications or medications that may affect glucose metabolism, (v) free from any form of illnesses or chronic diseases. Participants were excluded from the study if they had any form of cardiovascular, metabolic diseases, digestive ailments, if they smoked, were pregnant or lactating, and if they had any known allergies to pine bark extract.

### 2.2. Study Design

The study was an acute, placebo-controlled, single-blind, crossover, dose-response, exploratory study. The study required participants to come to the research facility at Massey University, Auckland, New Zealand for three separate visits with at least a 48 h washout period.

Briefly, at every visit, participants arrived at the facility after at least a 10 h overnight fast (water was allowed). The first study visit was a control visit whereby participants consumed a single placebo capsule containing microcrystalline cellulose. The second and third visits were treatment visits where patients consumed a single capsule of 50 and 400 mg of Enzogenol^®^, respectively. An OGTT was commenced at all three visits twenty minutes after consuming the capsule. Participants were given a bottle of 300 mL of glucose drink Carbotest (Fronine, Thermo Fisher Scientific, Victoria, Australia) containing 75 g of carbohydrates to consume within five minutes.

Capillary blood samples were obtained via finger pricking with a single use disposable lancet (Accu-Chek Safe T-Pro Plus) twenty minutes before the commencement of OGTT, and again at 0, 15, 30, 45, 60, 90, and 120 min during the OGTT. Blood glucose levels were immediately measured using a glucose meter (MediSense, Optium, Abbott, Auckland, New Zealand, 2.7–4.0% CV).

Participants were instructed to maintain a consistent diet without any dietary alterations throughout the duration of the study. They were also asked to abstain from alcohol and beverages such as teas, coffees, and energy drinks (both caffeinated and decaffeinated), all health supplements that might influence glucose metabolism, including any form of pine bark products, and strenuous activity during the 24h period before each visit. Compliance was checked at every visit by the researcher, including what they had eaten the previous day in order to ensure that they had been adhering to the dietary requirements regarding what they could eat or drink.

### 2.3. Treatments

Active capsules contained either 50 mg Enzogenol^®^ plus 350 mg microcrystalline cellulose or 400 mg Enzogenol^®^ prepared from the same batch of product. Placebo capsules contained 400 mg microcrystalline cellulose, which is commonly used as a filler in placebo capsules in clinical studies [38]. Both were supplied by ENZO Nutraceutical Limited (Paeroa, New Zealand). The doses chosen for this study were based on previous studies investigating the impact of pine bark (French Maritime) on glycaemic control in people with diabetes [39,40,41]. The doses chosen reflected the low and high doses used in previous studies with the focus on examining the acute hypoglycaemic impact of Enzogenol^®^ in healthy participants. The doses selected represented concentrations with no known toxicity [12]. The identical looking capsules were of opaque white appearance, concealing the visibility of the contents, and were produced to the standards of dietary supplement preparations and quality control in New Zealand.

### 2.4. Parameters of Glycaemic Response during the OGTT

The shape of the control glucose curve of each participant was determined using calculations employed by Tschritter et al. [23], Briefly, monophasic, biphasic, and triphasic curves were defined as Gluc_120_-Gluc_90_ < 0.25 mmol/L, Gluc_120_-Gluc_90_ >0.25 mmol/L and Gluc_90_-Gluc_60_ > 0.25 mmol/L, respectively. Participants exhibiting either a biphasic or triphasic glucose curve shape were grouped together as having a complex glucose curve shape.

The primary outcome, iAUC of postprandial glucose, was calculated during the OGTT from 0 to 120 min using the trapezoidal rule [42].

The %PG was defined by the percentage increment of 2hPG with respect to fasting blood glucose (FBG), using the formula [(2hPG-FBG)/FBG] × 100 [30].

The time to glucose peak was defined as the time point on the OGTT when the glucose level was highest. For complex glucose curve shapes (biphasic or triphasic), the time of the first glucose peak was considered [43].

The glucose peak value and the 2hPG were also recorded.

### 2.5. Statistical Analysis

A sample size calculation was performed on the change in postprandial capillary blood glucose based on a previous study conducted by our group on the acute responses of several plant extracts [44]. A minimum sample size of ten was required to detect a difference in postprandial glucose levels. However, to allow for potential withdrawals and the stratification of participants into two groups with either monophasic or complex glucose curve shapes, twenty-five participants were recruited.

A one-way factorial repeated measures ANOVA (95% confidence interval) was conducted to examine the effects of Enzogenol^®^ on postprandial blood glucose and glucose response indices in participants within each glucose curve shape classification. Statistical analysis was done comparing each dose (50 mg and 400 mg) with control.

Monophasic and complex groups were statistically compared with an Independent Student two-tailed *t*-test assuming equal variance. Analyses were performed with the SPSS software version 25 (IBM Corporation, New York, NY, USA). The results were reported as mean ± S.E.M.

## 3. Results

Twenty-five healthy participants (ten men and fifteen women, mean age 24.8 ± 0.8 years) were recruited into the study. All participants completed both the control and the 50 mg Enzogenol^®^ visit, and twenty also completed the 400 mg Enzogenol^®^ study visit. The demographics of this group and the comparison between the monophasic and complex groups are reported in Table 1. All participants were considered healthy based on their baseline data of mean BMI (21.2 ± 0.4 kg/m^2^), FBG (4.4 ± 0.1 mmol/L), HbA1c (33 ± 0 mmol/mol), blood pressure (systolic 109 ± 2, diastolic 67 ± 1 mmHg), and lipid profiles [45]. There was no significant difference in baseline characteristics between the monophasic and complex groups (*p* > 0.05).

The overall data obtained from all participants was first analysed before the stratification of participants into two groups. There was no significant reduction in the primary outcome mean glucose iAUC observed between control and intervention with 50 mg of Enzogenol^®^ (239.8 ± 19.2 vs. 276.8 ± 23.9 mmol/L·min, 13.4% reduction, *p* = 0.123), but a significant reduction between the control and 400 mg of Enzogenol^®^ (235.7 ± 16.5 vs. 299.5 ± 26.9 mmol/L·min, 21.3% reduction, *p* = 0.016). There was no significant dose response between the two doses 50 and 400 mg (*p* = 0.685).

**Figure 1:**
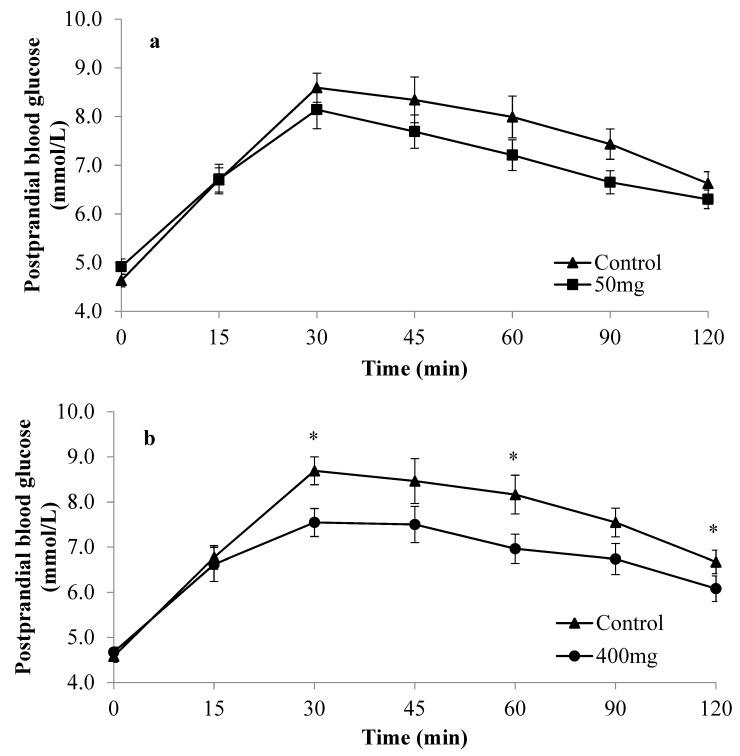
Mean postprandial glucose (±SEM) of participants with a monophasic curve shape during the control and treatment visits with (**a**) 50 and (**b**) 400 mg of Enzogenol^®^. * *p* < 0.05 for 400 mg Enzogenol^®^ compared to control.

**Figure 2:**
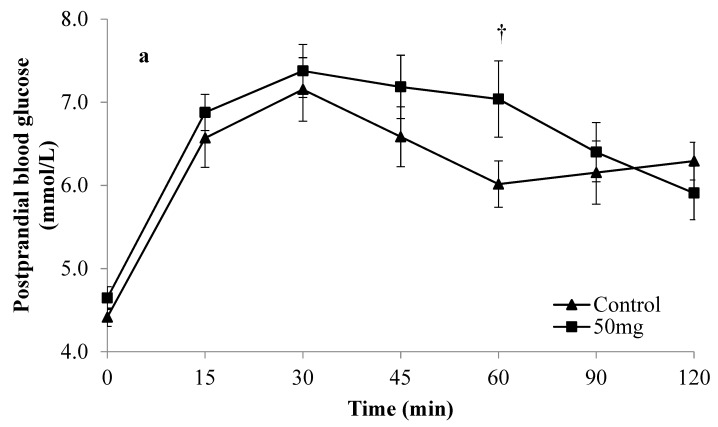

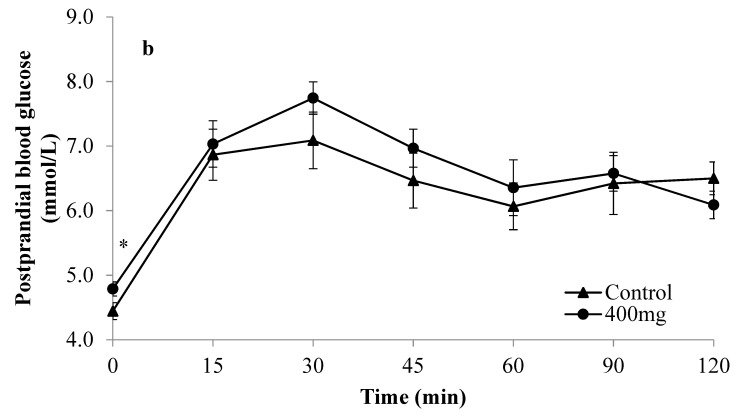
Mean postprandial glucose (±SEM) of participants with a complex glucose curve shape during control and treatment visits with (**a**) 50 and (**b**) 400 mg of Enzogenol^®^. ^†^ *p* < 0.05 for 50 mg Enzogenol^®^ compared to control, * *p* < 0.05 for 400 mg Enzogenol^®^ compared to control.

The participants were then classified into two distinct groups based on their glucose curve shapes at the control visit, either monophasic or complex group. Participants with monophasic shapes were shown to have a significantly higher mean glucose iAUC (*p* = 0.015) and glucose peak values (*p* = 0.021) than participants with complex shapes at the control visit, indicating the presence of two distinct groups with dissimilar glycaemic control. The effects of Enzogenol^®^ on postprandial glucose excursion differed depending on the type of postprandial glucose curve shapes participants exhibited at the control visit (Figure 1 and Figure 2). In Figure 1, involving participants with monophasic shapes, a consistent trend of reduced glucose levels was observed with both doses of Enzogenol^®^ compared to the control, although significantly lower glucose levels at 30, 60, and 120 min were only seen in the intervention with a 400 mg dose. Figure 2 illustrated more heterogeneous glucose excursions involving participants having complex shapes with no obvious trend after consuming both doses of Enzogenol^®^.

In the monophasic group, 33.4% of participants shifted from monophasic to having complex shapes (16.7% with biphasic and 16.7% with triphasic) after consuming 50 mg of Enzogenol^®^, and 54.6% of participants shifted to having complex shapes (18.2% with biphasic and 36.4% with triphasic) after consuming 400 mg of Enzogenol^®^.

With reference to Table 2, Enzogenol^®^ significantly reduced mean glucose iAUC in participants with a monophasic shape compared to control at both 50 mg (241.3 ± 20.2 mmol/L·min vs. 335.4 ± 34.0 mmol/L·min, 28.1% reduction, *p* = 0.034) and 400 mg of Enzogenol^®^ (249.3 ± 25.4 mmol/L·min vs. 353.6 ± 31.5 mmol/L·min, 29.5% reduction, *p* = 0.012). However, there was no significant dose response observed between 50 and 400 mg of Enzogenol^®^ within the monophasic group (*p* = 0.881), suggesting that a smaller dose of 50 mg was equally effective in reducing mean glucose iAUC of participants. In contrast, neither dose of Enzogenol^®^ significantly reduced the glucose iAUC in the complex group.

Mean %PG was reduced in both monophasic and complex groups compared to control. Treatment with 400 mg of Enzogenol^®^ in the monophasic group significantly reduced mean %PG compared to control (31.4% ± 7.9% vs. 47.5% ± 8.6%, 33.9% reduction, *p* = 0.010). No significant reduction in mean %PG was observed in this group after consuming 50 mg Enzogenol^®^. In the complex group, compared to control, the mean %PG was significantly reduced by both 50 mg (28.7% ± 8.2% vs. 43.4% ± 5.9%, 33.8% reduction, *p* = 0.012) and 400 mg of Enzogenol^®^ (27.7% ± 5.4% vs. 47.3% ± 7.2%, 41.4% reduction, *p* = 0.025).

There was no significant difference in mean time to glucose peak observed between control and all treatments in monophasic and complex groups. Most participants had their glucose peaking at 30 min during the OGTT, with a higher percentage of participants in the complex group having an earlier glucose peak at 15 min compared to monophasic group.

Within the monophasic group, a reduction in the mean glucose peak value was observed following both 50 and 400 mg of Enzogenol^®^, with the 400 mg dose being statistically significant (7.9 ± 0.3 vs. 8.9 ± 0.3 mmol/L, 11.2% reduction, *p* = 0.025) (Figure 1). There was no significant difference observed between control and treatments within the complex group.

Mean 2hPG was significantly reduced in the monophasic group treated with 400 mg of Enzogenol^®^ (6.1 ± 0.3 vs. 6.7 ± 0.3 mmol/L, 8.9% reduction, *p* = 0.027) (Figure 1). No significant changes were observed with 50 mg or in the complex group.

There was a lack of significant dose response observed between the two doses 50 and 400 mg in both the monophasic and complex groups in the primary and secondary outcome measurements (*p* > 0.05).

## 4. Discussion

Type 2 Diabetes Mellitus is an epidemic worldwide with an increasing number of people not adhering to a healthy diet and an active lifestyle. Because the progression of normoglycaemia towards impaired glycaemic control is a continuum, and even healthy, NGT individuals have been shown to have a certain degree of insulin resistance [25,29,30,31], it is becoming increasingly important to be able to classify individuals based on the varying degrees of dysglycaemia.

In the present study, a significant reduction in the primary outcome mean glucose iAUC was only seen for the 400 mg dose of Enzogenol^®^ (*p* = 0.016). However, when the participants were stratified by their baseline postprandial glucose curve shapes, it was shown that it was effective in reducing the postprandial glucose iAUC at both dose levels in participants with monophasic shapes. This group could be termed as responders as they responded well to the intervention by demonstrating improved glycaemic responses, such as a significantly reduced postprandial glucose iAUC, with additional improvements in other glucose response indices at the higher dose, 400 mg. This outcome was in contrast to participants with complex shapes who may then be termed as non-responders, as no significant improvements in the primary outcome glucose iAUC and other glucose response indices measured, except for %PG were evident.

To the best of our knowledge this is the first study to report on the glycemic-lowering properties of New Zealand pine bark extract in humans. Prior to this study, a diabetic mouse model fed with Enzogenol^®^ was reported to improve diabetes-related biomarkers with a reduction in HbA1c, insulin, and glucagon levels, and an elevation of hepatic AMP-activated protein kinase (AMPK) activity [46]. Pcynogenol^®^, a French maritime pine bark containing similar types of phenolic content but in different quantities compared to Enzogenol^®^, has been more extensively studied. Several chronic human trials have examined the impact of Pcynogenol^®^ in doses of 50–300 mg on glycaemic responses, such as FBG and HbA1c in T2DM participants [39,40,41]. All three studies showed a significant reduction in FBG and two studies showed a significant reduction in HbA1c. These studies elucidated the potential hypoglycaemic effects of pine bark, although they were conducted in T2DM participants, which differed from the present study with healthy participants.

The present study has employed the use of various glycaemic response indices during the 2h OGTT in relation to postprandial glucose curve shapes to investigate if Enzogenol^®^ would have an impact on various indicators of glycaemic control.

A study conducted by Schianca et al. elucidated that normoglycaemic individuals with lower %PG closer to FBG possessed higher insulin sensitivity and decreased insulin secretion compared to individuals with higher %PG [30]. Treatment with 400 mg of Enzogenol^®^ in the monophasic group and both doses, 50 and 400 mg, in the complex group significantly reduced %PG compared to control. The reduction in %PG could indicate the increased rate in returning postprandial blood glucose back to baseline FBG after two hours in both monophasic and complex groups with the consumption of Enzogenol^®^, which would suggest that there was a potential acute improvement in glycaemic control [47].

Research has shown that individuals exhibiting a monophasic glucose curve shape compared to those with a complex glucose curve shape (biphasic or triphasic) had a delayed rise in glucose with a later glucose peak, a higher glucose peak value, and a higher 2h postprandial glucose value, and are more likely to be at an increased risk of diabetes due to suboptimal glycaemic control [24,43]. Our observations agree with other studies where individuals with monophasic shapes exhibited higher postprandial glucose concentrations [23,24,25,26,27,28]. A delay in glucose peak time often indicates reduced insulin sensitivity and secretion and a rise in glucose typically observed in T2DM [48]. A time to glucose peak above 30 min was indicated to be a reproducible independent indicator of impaired glycaemic control [43,49]. Participants in the present study had mean glucose peaks occurring within 30 min on average for the complex group at control and with both treatments with Enzogenol^®^ (*p* > 0.05]. In contrast, the mean time to glucose peak in the monophasic group was slightly above 30 min for both control and treatments with Enzogenol^®^, although the time to glucose peak was shorter compared to control (*p* > 0.05).

The level of glucose peak has been associated with increased oxidative stress corresponding to the level of postprandial glucose toxicity [50]. Hulman et.al. concluded that a higher than normal glucose peak is more predictive of abnormality with insulin sensitivity than an absolute 2hPG value [51]. A recent cohort study further elucidated that individuals with a higher intermediate glucose value and a lower 2hPG value were associated with a higher risk of future diabetes than those with a higher 2hPG but a lower intermediate glucose value [52]. The present study found that 400 mg of Enzogenol^®^ was able to significantly reduce the glucose peak value compared to control in both the monophasic and complex groups (*p* = 0.025).

In the San Antonio Metabolism (SAM) study, “normal’ glucose-tolerant individuals having 2hPG values within the range of 6.7 to 7.8 mmol/L were found to exhibit a 40–50% decrease in beta-cell function compared to individuals with 2hPG lesser than 5.6 mmol/L [31]. Participants in the present study had mean 2hPG values that were lower than the at-risk range in all treatments except for participants in the monophasic group. Monophasic participants in the present study had a mean 2hPG value of 6.6 ± 0.2 mmol/L (this being obtained from capillary blood sample instead of plasma, as in the SAM study), which was close to the stated range indicative of a certain degree of beta-cell dysfunction. However, this higher 2hPG value was diminished with a subsequent treatment with Enzogenol^®^.

One of the merits of the study was the use of a robust crossover design where participants were their own control and underwent each of the treatments, and therefore a smaller sample size was required for the same level of significance. To our knowledge, this present study was one of the first intervention studies to classify normoglycaemic participants based on their degrees of impaired glycaemic control dependent on their glucose curve shapes, with the inclusion of useful glycaemic indices to determine if Enzogenol^®^ was effective in improving glycaemic responses. Furthermore, the potential modification of monophasic shapes into complex shapes with the consumption of Enzogenol^®^ was also examined. It was observed that about 33% of the monophasic participants changed to having complex shapes after consuming 50 mg of Enzogenol^®^, and about 55% of monophasic participants had complex shapes after consuming 400 mg of Enzogenol^®^. This might potentially alter T2DM risk based on the glucose shapes. This observation was in line with Manco et al., who concluded that individuals who persisted in having monophasic shapes and those who switched from having biphasic to monophasic shapes over a prolonged period had an increased risk of impaired glycaemic control [53]. A longer-term study is warranted to examine the persistence in shape alteration in relation to changes in glucose metabolism with the consumption of Enzogenol^®^.

Nevertheless, the study was not without limitations, which may include determining the glucose curve shapes based on the results of a single OGTT. Although, OGTT has been known as a sensitive test to detect mild disturbance in glucose metabolism and disposal [54], its reproducibility has been questioned. This was due mainly to differences in the rate of glucose absorption and insulin responses with varying degrees of glucose and insulin oscillations in individuals [55] and intra-individual variations [56,57,58], although one study found it to be repeatable in healthy individuals [59]. Hence, having repeated OGTT at baseline may improve reproducibility.

The classification of individuals into different glucose curve patterns might be simple and useful in an acute study with a smaller sample size, such as in the present study, but limitations may include the possible misclassification of individuals, especially if they exhibit heterogeneous glucose curves. However, we have classified our participants based on the calculations by Tschritter et al. [23] and verified with each postprandial glucose curve of each participant at the control visit to ensure there was no misclassification.

This study also only looked at postprandial glycaemic responses. Future studies should include insulin or C-peptide measurements to further our understanding of the effect of Enzogenol^®^ consumption on insulin secretion, insulin sensitivity, and beta-cell function (e.g., HOMA-IR, Matsuda index, and other measures). Future work may also explore the underlying mechanistic actions of Enzogenol^®^ in its glucose modulating effects in order to optimise on the dosage effective for lowering postprandial blood glucose. More investigation is warranted regarding the physiological metabolism of Enzogenol^®^ in humans through characterising phenolic metabolites from biological samples to investigate how the extract is being metabolised, absorbed, transformed, and excreted from the body. Future studies should also measure chronic glycaemic responses to Enzogenol^®^ over at least 8–12 weeks in both normo and hyperglycaemic participants. Future studies could also look into how Enzogenol^®^ can be incorporated into the diet, such as the effective dose, frequency, and sequence of consumption that helps to maintain a healthy glycaemic control and extend its functionality to individuals with varying degrees of impaired glycaemic control.

## 5. Conclusions

The present study in healthy participants shows that Enzogenol^®^ has hypoglycaemic effects, however, there was significant variation in inter-individual response, which appears to be driven by dissimilar individual glycaemic profile, with participants having monophasic glucose curve shapes showing greater improvements in postprandial glucose responses. Compared to healthy participants with complex glucose curve shapes, monophasic participants showed a significant improvement in mean glucose iAUC for both 50 and 400 mg doses of Enzogenol^®^, with a further reduction in glucose peak value, 2hPG, and %PG with 400 mg of Enzogenol^®^ compared to control. In contrast, glycaemic responses in participants with complex glucose curve shapes were not altered with the ingestion of Enzogenol^®^ except for significant reduction in %PG values. Although Enzogenol^®^ appears to show hypoglycaemic potential, future studies are warranted to examine the effect on other measures, such as insulin, and in other population groups, such as those with prediabetes or T2DM.

## Figures and Tables

**Table 1:** Baseline characteristics of participants (*n* = 25) classified as having either a monophasic (*n* = 12) or complex (*n* = 13) glucose curve shape during the control visit.

Characteristics	Mean ± SEM (All)	Range (Min to Max)	Mean ± SEM (Monophasic Group)	Mean ± SEM (Complex Group)	*p*(Monophasic vs. Complex Shape)
N	25	NA	12	13	-
Gender (M/F)	10/15	NA	3/9	7/6	-
Age (years)	24.8 ± 0.8	20–33	25.4 ± 1.1	24.2 ± 1.2	0.44
BMI (kg/m^2^)	21.2 ± 0.4	18.1–25.3	21.2 ± 0.6	21.2 ± 0.5	1.00
FBG (mmol/L)	4.4 ± 0.1	3.6–5.2	4.5 ± 0.1	4.3 ± 0.1	0.55
HbA1c (mmol/mol)	33 ± 0	29–38	33 ± 1	34 ± 1	0.24
SBP (mm Hg)	109 ± 2	89–133	111.7 ± 4.4	107 ± 2	0.30
DBP (mm Hg)	67 ± 1	47–77	65.6 ± 2.4	68 ± 2	0.45
TC (mmol/L)	4.09 ± 0.12	3.06–5.27	4.07 ± 0.13	4.10 ± 0.20	0.89
TG (mmol/L)	1.15 ± 0.13	0.56–3.71	1.30 ± 0.25	1.02 ± 0.11	0.30
HDL-C (mmol/L)	1.50 ± 0.08	0.98–2.49	1.47 ± 0.11	1.54 ± 0.11	0.67
LDL-C (mmol/L)	2.06 ± 0.09	1.23–2.93	2.01 ± 0.13	2.10 ± 0.12	0.60
Non-HDL-C (mmol/L)	2.58 ± 0.09	1.88–3.32	2.60 ± 0.13	2.57 ± 0.13	0.87
TC/HDL-C ratio	2.82 ± 0.10	1.8–4.1	2.89 ± 0.17	2.75 ± 0.12	0.49

Values are means (± SEM). Abbreviations: BMI: body mass index; FBG: fasting blood glucose; HbA1c: glycated haemoglobin A1c; SBP: systolic blood pressure; DBP: diastolic blood pressure; TC: total cholesterol; TG: triglycerides; HDL-C: high-density lipoprotein cholesterol; LDL-C: low-density lipoprotein cholesterol.

**Table 2:** Parameters of glycaemic response in participants classified as having either a monophasic or complex glucose curve shape at control visit and treatments with 50 and 400 mg of Enzogenol^®^.

Parameters of Glycaemic Response	Monophasic Group						Complex Group					
	Control Paired to 50 mg of Enzogenol^®^	50 mg of Enzogenol^®^	*p*	Control Paired to 400 mg of Enzogenol^®^	400 mg of Enzogenol^®^	*p*	Control Paired to 50 mg of Enzogenol^®^	50 mg of Enzogenol^®^	*p*	Control Paired to 400 mg of Enzogenol^®^	400 mg of Enzogenol^®^	*p*
*n*	12		-	11		-	13		-	9		-
Gender (M/F)	3/9		-	3/8		-	7/6		-	5/4		-
Mean glucose iAUC (mmol/L·min)	335.4 ± 34.0 ^†^	241.3 ± 20.2	0.034 *	353.6 ± 31.5 ^‡^	249.3 ± 25.4	0.012 *	222.7 ± 26.6 ^†^	238.4 ± 32.7	0.392	233.5 ± 36.4 ^‡^	219.1 ± 19.5	0.614
Mean %PG	44.9 ± 8.3	28.9 ± 3.8	0.083	47.5 ± 8.6	31.4 ± 7.9	0.010 *	43.4 ± 5.9	28.7 ± 8.2	0.012*	47.3 ± 7.2	27.7 ± 5.4	0.025 *
Mean time to glucose peak (min)	35.0 ± 2.8	32.5 ± 2.5	0.504	35.5 ± 3.0	32.7 ± 4.0	0.441	28.8 ± 2.7	28.8 ± 4.0	1.000	28.3 ± 3.0	26.7 ± 4.9	0.729
Mean glucose peak value (mmol/L)	8.8 ± 0.3^†^	8.3 ± 0.4	0.177	8.9 ± 0.3^‡^	7.9 ± 0.3	0.025 *	7.7 ± 0.3 ^†^	8.2 ± 0.4	0.315	7.8 ± 0.3 ^‡^	8.0 ± 0.3	0.531
Mean 2hPG (mmol/L)	6.6 ± 0.2	6.3 ± 0.2	0.171	6.7 ± 0.3	6.1 ± 0.3	0.027 *	6.3 ± 0.2	5.9 ± 0.3	0.149	6.5 ± 0.3	6.1 ± 0.2	0.224

* Significant difference between control and 50 or 400 mg of Enzogenol^®^ within each glucose curve shape group (*p* < 0.05); ^†^ Significant difference in control visit values between monophasic and complex glucose curve shape groups at 50 mg of Enzogenol^®^ (*p* < 0.05); ^‡^ Significant difference in control visit values between monophasic and complex glucose curve shape groups at 400 mg of Enzogenol^®^ (*p* < 0.05); Abbreviations: iAUC: incremental area under the curve of postprandial glucose; %PG: percentage increment of postprandial glucose; 2hPG: 2h postprandial glucose at 120 min of the oral glucose tolerance test (OGTT).
